# Bilateral corneal perforation caused by neurotrophic keratopathy associated with leprosy: a case report

**DOI:** 10.1186/s12886-022-02265-6

**Published:** 2022-01-29

**Authors:** Satoshi Iraha, Shoko Kondo, Takefumi Yamaguchi, Toshihiro Inoue

**Affiliations:** 1 Department of Ophthalmology, National Sanatorium Kikuchi Keifuen, 3796 Sakae, Koshi, Kumamoto 8611113 Japan; 2 Department of Ophthalmology, Kumamoto University Hospital, Kumamoto, Japan; 3 Department of Ophthalmology, Tokyo Dental College Ichikawa General Hospital, Chiba, Japan

**Keywords:** Leprosy, Neurotrophic keratopathy, Corneal perforation, Hansen’s disease, Complication

## Abstract

**Background:**

Neurotrophic keratopathy (NK) is a rare degenerative corneal disease caused by damage to the trigeminal nerve. We hereby describe a severe case with bilateral corneal perforation due to leprosy (Hansen’s disease)-associated NK.

**Case presentation:**

An 89-year-old man with a history of leprosy treated 40 years previously in our sanatorium developed bilateral corneal perforation due to NK. He had a history of bilateral persistent epithelial defects and bacterial keratitis. Although epithelialization was obtained with the use of autologous serum eye drops, progressive corneal thinning concomitant with stromalysis led to bilateral perforation. Over one month treatment with topical antibiotics, anti-inflammatory and lubricants resulted in healing of the epithelial defects and corneal perforations. A Cochet-Bonnet esthesiometer demonstrated a total absence of corneal sensation in both eyes.

**Conclusions:**

The present case indicated the irreversible nerve damage due to leprosy that had been cured 23 years ago, which can progress over the years and cause bilateral corneal perforations.

## Background

Neurotrophic keratopathy (NK) is a rare degenerative corneal disease caused by damage to the trigeminal nerve. Dysfunction of the trigeminal nerve results in reduced corneal sensitivity and impairment of corneal epithelial wound healing, leading to persistent epithelial defects, ulceration, stromalysis, and perforation. Various conditions and diseases, including herpetic keratitis, diabetes mellitus, and chemical and surgical injury can cause NK by damaging trigeminal nerves [1, 2].

Leprosy, also known as Hansen’s disease, is a chronic neurocutaneous infectious disease caused by *Mycobacterium leprae* [3]. This bacterium mainly targets Schwann cells in the peripheral nerves, and sometimes tends to involvement of the branches of the cranial nerves, mainly affecting the facial and trigeminal nerves [4, 5]. With the development of dapsone in the 1940s and the World Health Organization’s recommendation of multidrug therapy in 1981, leprosy has become a curable disease. Although patients are considered cured after treatment, they may still suffer neuronal and ocular complications as a result of nerve damage.

We have long cared for leprosy patients in our sanatorium, which is the largest national leprosy sanatorium in Japan and was established in 1909. Dry eye, pterygium, lid abnormalities and reduced corneal sensation have been reported as ocular complication of leprosy [6, 7]. However, there has been no reports on corneal perforations associated with NK due to leprosy. Here, we report the first case of bilateral corneal perforation due to severe leprosy-induced NK which developed a long time after completion of leprosy treatment.

## Case presentation

An 89-year-old man, followed over 30 years for leprosy-associated ocular complications, presented to our sanatorium for monthly examination. Two years before this presentation, he had bilateral persistent epithelial defects and bacterial keratitis. Although epithelialization was obtained with the use of autologous serum eye drops, corneal thinning of left eye progressed to perforation in the peripheral region at 9 o’clock position. The perforation closed by the treatment with eye drops and ointments. He also had a prior history of bilateral lower eyelid retraction for lagophthalmos caused by facial nerve paralysis due to leprosy, pterygium, and cataract surgery. There were no other ocular infections (HSV and HZV) and ocular injury. Fundoscopic examination with +20D lens and optical coherence tomography (OCT) scan evaluation showed fundus of the both eyes were normal.

He was first diagnosed with lepromatous leprosy (LL) at the age of 15 years and it was cured after treatment with dapsone at the age of 65 years although he had glove and stocking anesthesia, pain less ulcer and contractures of hands and feet. He had no additional relevant medical history, including genetic disease and systemic disease (diabetes and central nervus system disease) that can cause NK.

**Fig. 1:**
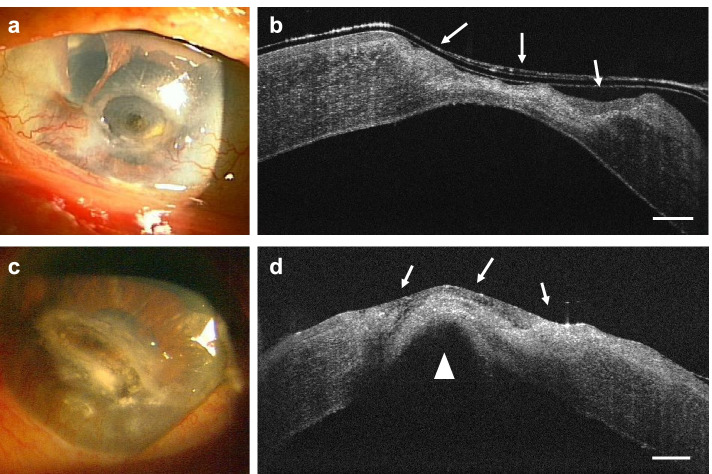
Slit lamp photograph and Cross-sectional anterior segment optical coherence tomography (AS-OCT) of the left eye (**a, b**) and right eye (**c**, **d**). **a** Slit lamp photograph of the patient’s left eye showing corneal stroma thinning. Corneal neovascularization can be seen in areas of surgical scarring arising from pterygium and pseudopterygium surgery at the 3 and 9 o’clock positions, respectively. An iris defect, which occurred during phacoemulsification surgery, can be seen at the 11 o’clock position. **b** AS-OCT image of the left eye. Arrows indicate an area of stromal thinning lacking corneal epithelium. **c** Slit lamp photograph of the patient’s right eye showing corneal perforation. **d** AS-OCT image of the right eye. Arrows indicate an irregular appearance of the stroma and epithelial defect. The arrowhead indicates a high-intensity region beneath the cornea. Scale bars: 500 μm

Examination demonstrated best-corrected visual acuity (BCVA) of 20/80 in the right eye and 20/2000 in the left eye. Although he had no pain, a corneal ulcer was detected in the central region of the left eye on slit-lamp examination (Fig. 1a). Cross-sectional anterior segment OCT (AS-OCT) showed stromal thinning (Fig. 1b). Fundus examination of the left eye showed neither retinal detachment nor vitreous hemorrhage. Corneal scraping for bacterial and fungal culture was negative. Because of the absence of corneal sensation judged by lack of pain against corneal ulcer, he was diagnosed with persistent epithelial defect due to NK. To prevent complications of corneal infection, he was treated with 0.5% cefmenoxime and 0.1% fluorometholone four times daily, 0.3% ofloxacin ointment twice daily, and 0.1% purified sodium hyaluronate eye drops six times daily in the left eye. Two months later, the epithelial defect was closed.

Three months later, he presented with blurred vision in right eye without pain. BCVA in his right eye was hand motion. Corneal perforation was detected in the central region of the right eye (Fig. 1c). AS-OCT showed the stromalysis and fibrin beneath the cornea (Fig. 1d). Because of the corneal perforation, we couldn’t check fundus of right eye. Treatment with 2% glutathione eye drops four times daily, 0.1% fluorometholone three times daily, 0.1% purified sodium hyaluronate eye drops five times daily and 0.3% ofloxacin ointment three times daily successfully addressed the absence of epithelium. Three months later, a corneal sensitivity test using a handheld Cochet–Bonnet esthesiometer (Handaya Co., Ltd., Tokyo, Japan) demonstrated an absence of sensitivity; the filament length of five corneal quadrants was < 5 mm in both eyes. Schirmer’s test showed almost normal tears in both eyes, with strip wetting of 14 mm in the right eye and 20 mm in the left eye. Patient continued to be closely monitored with treatment.

## Discussions and conclusions

Systemic diseases such as diabetes, multiple sclerosis, and leprosy may decrease sensory nerve function or damage sensory fibers leading to corneal anaesthesia and may lead to the development of NK [8]. We report a case of severe leprosy-associated NK leading to bilateral corneal perforation. Although several studies have shown that patients continue to develop new ocular complications after successful treatment of leprosy [9–11], to our knowledge there has been no previous report of severe NK associated with cured leprosy.

Leprosy is one of the causes of corneal hyposensitivity, but deep corneal hyposensitivity approaching anesthesia is rare [12]. Karaçorlu et al. used a Cochet-Bonnet esthesiometer to examine corneal sensitivity in 286 eyes of leprosy patients, and found that 10.5% (30 of 286 eyes) of patients responded to a filament length < 30 mm, which was considered advanced hyposensitivity, while only 0.6% (2 of 286 eyes) responded to a filament length < 5 mm [12].

In this case, not only the complications of leprosy, aging and the type of leprosy might affect the progression of NK. Aging itself is a risk factor for NK [1] and also a risk factor for ocular complications in leprosy patients even after completion of multidrug therapy [10, 11]. In our sanatorium, the average age of cured patients increased to 85.3 years old in 2021. Careful attention must be paid to the possible development of NK. The classification of this patient was LL type. LL type is characterized by the absence of a specific response with uncontrolled proliferation of leprosy bacilli and extensive infiltration of the nerves [3]. Daniel at al. showed elderly and LL type are associated with increased morbidity of ocular complications [7]. We should pay attention to the type of leprosy.

Corneal nerve regeneration is well-known in NK after herpetic keratitis [13], diabetes [14] and refractive surgeries [15]. In contrast, there has been no reports on corneal nerve regeneration in NK due to post-brain surgery, multiple sclerosis, and leprosy. Recent studies have shown that nerve growth factor or platelet-rich plasma improve skin sensation in leprosy peripheral neuropathy [16, 17]. Because severe leprosy-associated NK can progress and cause bilateral corneal perforation, future studies on corneal nerve regeneration therapy would be valuable.

In conclusion, we should know that leprosy can cause NK even years after the complete remission of leprosy. In patients who have already sustained peripheral nerve damage resulting in sensory and motor impairment, it is taxing to struggle with new eye complications in later life. This case showed that careful follow-up should be continued to prevent the development of severe ocular complications, such as bilateral corneal perforation due to persistent NK, even decades after leprosy has been cured.

## Data Availability

Data sharing is not applicable to this article as no datasets were generated or analyzed during the current study.

## Abbreviations

NK: neurotrophic keratopathy
LL: lepromatous leprosy
BCVA: best-corrected visual acuity
AS-OCT: anterior segment optical coherence tomography
